# Lenvatinib-Associated Posterior Reversible Encephalopathy Syndrome in a Patient With Metastatic Clear Cell Renal Cell Carcinoma: A Case Report

**DOI:** 10.7759/cureus.111672

**Published:** 2026-06-28

**Authors:** Nasong A Luginaah, Seth Climans, Aquila Akingbade

**Affiliations:** 1 Schulich School of Medicine and Dentistry, Western University, London, CAN; 2 Department of Oncology, London Health Sciences Centre, London, CAN; 3 Department of Clinical Neurological Sciences, London Health Sciences Centre, London, CAN; 4 Division of Radiation Oncology, London Health Sciences Centre, Western University, London, CAN

**Keywords:** clear-cell renal carcinoma, drug-induced neurotoxicity, lenvatinib, posterior reversible encephalopathy syndrome (pres), reversible posterior leukoencephalopathy syndrome (rpls), vegfr tyrosine kinase inhibitors

## Abstract

Posterior reversible encephalopathy syndrome (PRES) is a rare but serious neurologic complication associated with vascular endothelial growth factor receptor (VEGFR) tyrosine kinase inhibitors, including lenvatinib. We report a case of PRES in a 63-year-old woman with metastatic clear cell renal cell carcinoma treated with pembrolizumab and lenvatinib for isolated pancreatic metastases. Two days after lenvatinib initiation, she developed headaches, confusion, visual hallucinations, and a generalized tonic-clonic seizure in the setting of severe hypertension. Brain magnetic resonance imaging demonstrated bilateral parieto-occipital vasogenic edema consistent with PRES. Lenvatinib was discontinued, and she was treated with antihypertensive and antiepileptic therapy, with complete clinical recovery within two weeks with radiographic resolution on follow-up imaging. Pembrolizumab was maintained as monotherapy without PRES recurrence. This case highlights the importance of prompt recognition, drug discontinuation, blood pressure control, and supportive management in patients who develop neurologic symptoms shortly after starting VEGFR tyrosine kinase inhibitors.

## Introduction

Posterior reversible encephalopathy syndrome (PRES) is a rare but potentially serious neurologic complication associated with vascular endothelial growth factor receptor (VEGFR) tyrosine kinase inhibitors, including lenvatinib [1]. It is typically characterized by headaches, visual disturbances, seizures, encephalopathy, and radiographic evidence of vasogenic edema, most commonly in the parieto-occipital regions on MRI [2,3]. Although this toxicity is rare, early recognition is important because prompt withdrawal of the offending agent and supportive management often lead to clinical and radiographic recovery [4].

Lenvatinib is a multi-targeted VEGFR tyrosine kinase inhibitor selected for this patient as per the established CLEAR trial regimen for metastatic clear cell renal cell carcinoma (RCC) [5]. It works by binding to and blocking VEGF receptors on endothelial cells, thereby inhibiting the signaling cascades responsible for tumor blood vessel formation [6]. However, this same mechanism of action has systemic consequences: VEGF signaling is critical not only for tumor angiogenesis but also for maintaining normal endothelial cell survival, vascular tone, and blood-brain barrier integrity throughout the body [1,4]. When VEGF signaling is pharmacologically suppressed, endothelial dysfunction can develop systemically, impairing the brain's capacity to autoregulate cerebral blood flow. This breakdown in cerebrovascular autoregulation, particularly in the setting of acute hypertension, a well-recognized adverse effect of lenvatinib, can lead to forced hyperperfusion, vasogenic edema, and ultimately PRES [1,4].

Lenvatinib is an established systemic therapy option in metastatic clear cell RCC, but its use may be complicated by adverse effects including hypertension, proteinuria, thromboembolic events, hypothyroidism, and, rarely, PRES [7-9]. The pathophysiology of PRES in this setting is not fully understood, but inhibition of the VEGF pathway is thought to contribute to endothelial dysfunction, impaired cerebral autoregulation, and vasogenic edema [1].

Published reports of lenvatinib-associated PRES remain limited, particularly in RCC. We report a case of PRES occurring shortly after initiation of lenvatinib plus pembrolizumab in a 63-year-old woman with metastatic clear cell RCC.

## Case presentation

A 63-year-old woman with a remote history of clear cell RCC underwent left radical nephrectomy in 2014 for pT3a Nx disease (American Joint Committee on Cancer 7th edition) [10] with negative surgical margins. She remained disease-free until 2024, when surveillance imaging demonstrated multiple pancreatic lesions. Endoscopic ultrasound-guided biopsy confirmed metastatic clear cell RCC. Given the isolated pancreatic metastases and favorable International Metastatic RCC Database Consortium risk profile, she was started on pembrolizumab (200 mg intravenously every three weeks) and then lenvatinib (20 mg orally daily) per the CLEAR trial regimen [5]. Her baseline blood pressure was 120/79 mmHg. The patient had no additional recognized risk factors for PRES, including diabetes, vascular disease, autoimmune disease, prior seizures or neurologic disorders, or use of medications associated with PRES risk, such as corticosteroids or anticoagulants.

Ten days after her first dose of pembrolizumab, she reported mild arthralgia and a small oral aphthous ulcer. Laboratory assessment revealed biochemical hyperthyroidism (thyroid-stimulating hormone (TSH) 0.01 mIU/L (normal range 0.27-4.2 mIU/L), free T4 71 pmol/L (normal range 12-22 pmol/L), and free T3 24 pmol/L (normal range 3.1-6.8 pmol/L)), attributed to pembrolizumab. Lenvatinib was initiated the same day.

Two days after initiating lenvatinib, she presented to an outside emergency department after developing a headache and malaise. She was found to have new hypertension (160/92 mmHg). She was discharged home from the emergency department.

Over the following 10 days, her headache worsened and was accompanied by confusion and visual hallucinations described as seeing “red dots.” She subsequently returned to the emergency department with a reduced level of consciousness and worsened hypertension (187/85 mmHg), then experienced a witnessed tonic-clonic seizure in the emergency department. On admission, she was somnolent but responsive to pain, without focal neurologic deficits. Cardiorespiratory examination was normal. A comprehensive metabolic panel revealed normal electrolytes and renal function, with a mildly elevated alanine aminotransferase level (152 U/L; upper limit of normal ≤35 U/L).

A brain MRI during admission demonstrated bilateral cortical and subcortical T2/FLAIR hyperintensities in the parieto-occipital lobes without restricted diffusion or enhancement, consistent with PRES (Figure 1A). There were no intracranial metastases or hemorrhage. As other etiologies, including metabolic derangements and CNS metastases, were excluded, lenvatinib-associated PRES was suspected, given the temporal association with lenvatinib initiation and the presence of severe acute hypertension, seizure, encephalopathy, and characteristic MRI findings. Upon diagnosis, lenvatinib was immediately discontinued. She was treated with ramipril 5 mg twice daily, resulting in prompt normalization of blood pressure. Levetiracetam 500 mg twice daily was initiated for seizure prevention and discontinued after approximately one month. Supportive therapy included intravenous fluids and rest. Her neurologic symptoms gradually improved, and she returned to baseline function within two weeks. In accordance with standard seizure precautions, the patient was advised to refrain from driving for a minimum of three months following her seizure. Follow-up MRI demonstrated near-complete resolution of the parieto-occipital abnormalities, confirming reversibility (Figure 1B). The clinical and radiographic reversibility further supported the diagnosis of PRES.

**Figure 1:**
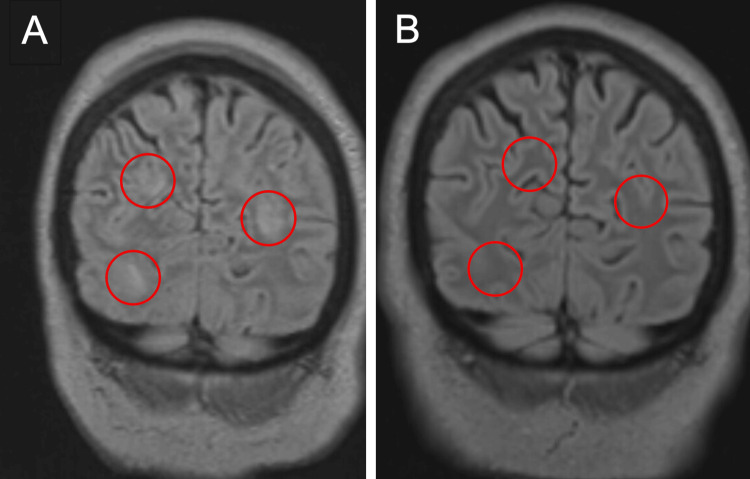
Brain MRI demonstrating PRES in a 63-year-old woman who developed PRES following lenvatinib initiation for metastatic clear cell RCC (A) Coronal T2/FLAIR image showing bilateral parieto-occipital vasogenic edema consistent with PRES. (B) Follow-up coronal T2/FLAIR image five weeks after lenvatinib discontinuation showing near-complete resolution, confirming reversibility. MRI: magnetic resonance imaging, PRES: posterior reversible encephalopathy syndrome, RCC: renal cell carcinoma

One month after admission, she developed autoimmune hypothyroidism (TSH 67.8 mIU/L, undetectable free T4), which was managed with levothyroxine 100 µg daily. Once clinically recovered, pembrolizumab monotherapy was resumed on a six-week schedule. She tolerated treatment without recurrence of neurologic symptoms. Over the following year, she remained clinically stable on pembrolizumab monotherapy with no recurrent seizures, encephalopathy, or PRES. Serial CT imaging of the chest, abdomen, and pelvis demonstrated stable pancreatic metastases with no new disease. The case timeline is summarized in Figure 2.

**Figure 2:**
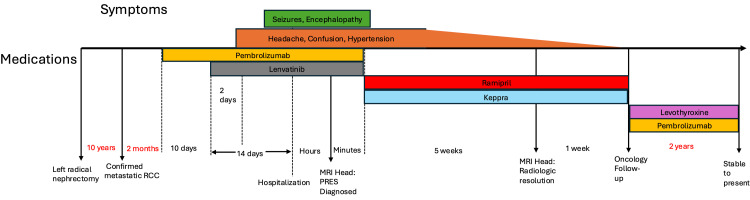
Graphical timeline of the clinical course from initial nephrectomy through PRES onset, management, and resumption of pembrolizumab monotherapy in a 63-year-old woman with lenvatinib-associated PRES and metastatic clear cell RCC Timeline is not to scale. yrs: years, mos: months, wks: weeks, wk: week, d: days, PRES: posterior reversible encephalopathy syndrome, RCC: renal cell carcinoma

## Discussion

PRES is a rare but recognized complication of VEGFR tyrosine kinase inhibitors, including lenvatinib. In the CLEAR trial, PRES was reported in 0.6% of patients treated with lenvatinib plus pembrolizumab and in 0.3% of those treated with lenvatinib plus everolimus; similarly, post-marketing data suggest an incidence of approximately 0.3% with lenvatinib monotherapy [5].

In this case report, the close temporal relationship between initiation of lenvatinib and onset of headache, encephalopathy, severe hypertension, and seizure, together with characteristic MRI findings and subsequent clinical and radiographic resolution after lenvatinib discontinuation, is consistent with lenvatinib-associated PRES as the most likely diagnosis. Although the pathophysiology remains incompletely understood, inhibition of the VEGF pathway is thought to promote endothelial dysfunction, hypertension, impaired cerebral autoregulation, and disruption of the blood-brain barrier, resulting in vasogenic edema [1].

To identify additional cases of lenvatinib-associated PRES, we conducted a systematic search of PubMed, Web of Science, Scopus, and EMBASE for reports that specifically describe PRES associated with lenvatinib; studies examining PRES associated with other TKIs were excluded. We identified seven published reports, summarized in Table 1. Most reported cases presented with hypertension and neurologic symptoms such as headache, confusion, visual disturbances, or seizures. Symptom onset ranged from hours to several weeks after lenvatinib initiation, highlighting the need for continued vigilance, particularly early in therapy.

**Table 1:** Characteristics of all previously documented lenvatinib-associated PRES cases * These are reported cases with no other information released. ARB: angiotensin receptor blocker, BP: blood pressure, CCB: calcium channel blocker, DL: dyslipidemia, F: female, HCC: hepatocellular carcinoma. HTN: hypertension, IV: intravenous, LEAP: lenvatinib and pembrolizumab combination, M: male, PMH: past medical history, PO: per oral, PRES: posterior reversible encephalopathy syndrome, PTC: papillary thyroid cancer, RCC: renal cell carcinoma, T2DM: type 2 diabetes mellitus

Author, year of publication, citation	Age, sex	Oncologic diagnosis	Relevant comorbidities	Lenvatinib regimen and time to PRES	Presentation	MRI findings	Management	Outcomes
Chae et al., 2018 [11]	58, F	Recurrent PTC; new breast cancer; liver metastases of unknown origin	Tumour fever	First dose of lenvatinib triggered PRES within hours, unknown dose	Generalized tonic-clonic seizures, altered mental status, and fever	Posterior-predominant multifocal white matter edema with patchy enhancement (including frontal involvement) and incidental small infarct; consistent with PRES	Lenvatinib discontinued; supportive blood pressure control	Patient discharged but died one week later due to cancer progression. Prior to death, repeat MRI showed no PRES
Osawa et al., 2018 [12]	66, F	Right PTC (pT3 N1b M0), and left ATC (pT4b N0 M0) (AJCC)	T2DM, DL, angina, and transient cerebral ischemia, HTN (controlled)	Started at 24 mg daily. Decreased to 20 mg after nine days due to HTN. Patient had visual field defect 10 days after dose reduction, and dose was reduced to 14 mg. After 13 days of 14 mg, PRES onset occurred	HTN, visual field defects, altered mental status	Bilateral occipital white matter T2/FLAIR hyperintensities consistent with PRES	Lenvatinib discontinued; treated with angiotensin receptor blocker and calcium channel blocker for blood pressure control	Complete clinical and radiographic resolution by day eight. Lenvatinib resumed at reduced dose (8 mg/day); no PRES recurrence; patient died 12 months later from carotid rupture; MRI prior to death showed no PRES
Abhishek et al., 2022 [13]	70, F	Metastatic PTC	HTN	10 mg daily then increased to 14 mg. Patient was on lenvatinib for four months before PRES	Hypertension, severe headache, and left arm weakness	Diffuse T2/FLAIR hyperintensities involving periventricular white matter, thalami, brainstem, and centrum semiovale with chronic pontine lacunar infarct, all suggestive of atypical PRES	Lenvatinib discontinued; corticosteroids administered with clinical improvement	Developed grade four lenvatinib toxicity, including atypical PRES and nephrotic syndrome. On treatment with steroids for nephrotic syndrome and pazopanib for thyroid cancer, which she is tolerating well. The patient has no residual neurologic deficits, suggesting PRES reversal
Tseng et al., 2022 [1]	40, M	Olfactory neuroblastoma	HTN (well-controlled)	10 mg daily for seven months before neurologic event; after stopping for ~two weeks continued for four weeks before PRES	Altered mental status, seizures, HTN	Residual frontal abscess with hydrocephalus/ventriculitis; new bilateral parieto-occipital T2/FLAIR hyperintense lesions consistent with PRES	Lenvatinib discontinued; blood pressure managed with amlodipine and labetalol	Lenvatinib resumed with no recurrence of PRES. Resolution on follow-up MRI
Buenaventura et al., 2023 [14]	65, F	Metastatic PTC (T4aN1bM0) (AJCC)	HTN, obesity	24 mg/day for four weeks before PRES	Headache, tonic-clonic seizures, altered mental status, HTN	Posterior-predominant cortico-subcortical T2/FLAIR hyperintensities without diffusion restriction, consistent with PRES	Lenvatinib discontinued; levetiracetam initiated for seizures, symptomatic BP control	Blood pressure normalized by day seven; prolonged hospitalization for refractory headache, resolved with pericranial blockade. At four-week follow-up, lenvatinib restarted at 10 mg and increased to 14 mg with no adverse events reported. At three months, patient asymptomatic; MRI showed complete resolution
Matsuura et al., 2022 [15]	53, F	Endometrial cancer (IVB)	N/A	LEAP protocol (20 mg lenvatinib PO daily and 200 mg pembrolizumab IV every three weeks). Developed PRES on day 18	Altered mental status, seizures, HTN	Mild isolated left occipital T2/FLAIR hyperintensity without evidence of infarction	LEAP discontinued; IV levetiracetam for seizures and symptomatic blood pressure control	Findings favored PRES over encephalitis; symptoms were thought to be due to lenvatinib rather than pembrolizumab. Pembrolizumab resumed; CT showed metastatic disease
Chen et al., 2025 [16]	72, F	HCC (T2N0M0) (AJCC)	N/A	8 mg/day for seven weeks before PRES	Altered mental status, HTN, headache	Bilateral periventricular and basal ganglia T2/FLAIR hyperintensities consistent with vasogenic edema (PRES)	Lenvatinib discontinued; blood pressure controlled with IV urapidil	Follow-up MRI showed reduced bilateral white matter signal abnormalities with rapid clinical improvement; long-term outcomes not reported
Luginaah et al., 2026	63, F	Clear cell RCC (pT3a NX)	N/A	20 mg/day for two days before symptoms, 14 days before presentation	Headache, confusion, HTN, seizure, encephalopathy	Bilateral parieto-occipital vasogenic edema	Lenvatinib discontinued, levetiracetam used for seizures, ramipril for BP management	Restarted pembrolizumab, stable disease with no recurrence to present
CLEAR trial case #1*	-	-	-	-	-	-	-	-
CLEAR trial case #2*	-	-	-	-	-	-	-	-

Although no formal diagnostic criteria for PRES exist, most cases are confirmed with a brain MRI. Findings are typically T2/FLAIR white matter hyperintensities, most often involving the parieto-occipital regions, although atypical distributions have also been described [3].

While the temporal association with lenvatinib initiation supports a potential drug-related etiology, alternative causes were considered. Pembrolizumab-associated immune-related encephalitis was considered less likely given the timing of symptom onset, the absence of inflammatory MRI findings, the complete resolution after lenvatinib discontinuation, and the lack of recurrence following pembrolizumab rechallenge. Unlike PRES, immune checkpoint inhibitor-associated encephalitis, such as pembrolizumab-associated immune-related encephalitis, typically occurs later in the course of treatment and may present with inflammatory features, including pleocytosis in the CSF or elevated protein levels [17]. Brain metastases, metabolic abnormalities, and electrolyte disturbances were excluded through imaging and laboratory assessment. Acute hypertension was also considered secondary to lenvatinib, given her normal baseline blood pressure and onset of hypertension after treatment initiation.

Among the previously reported cases summarized in Table 1, six of seven patients were female; including the present case, seven of eight documented cases of lenvatinib-associated PRES have occurred in women. Although no established sex-specific risk has been described for TKI-induced PRES, this pattern is consistent with the broader female predominance reported in PRES. This sex predilection has been attributed to the higher prevalence of recognized risk factors in women, including autoimmune diseases and pregnancy-related endothelial injury, such as preeclampsia and eclampsia [2,18]. While PRES most commonly affects women of reproductive age [2], all female patients in the included case reports were likely postmenopausal. Prior studies have demonstrated a decline in endothelial function during the perimenopausal period, with progressive worsening associated with increasing duration of estrogen deficiency [19].

Primary management of lenvatinib-associated PRES centers on immediate discontinuation of lenvatinib and supportive care, particularly antiepileptic and antihypertensive therapies, as indicated. This approach was used in our patient and in all seven reported cases. Clinical outcomes are generally favorable when PRES is recognized early, with most reports describing neurologic recovery and radiographic improvement or resolution on follow-up imaging. EEG and CSF analysis were not performed in this case. The diagnosis was supported by the characteristic MRI findings, clinical presentation, and temporal association with lenvatinib initiation, with complete resolution following drug discontinuation.

An additional feature of this case is that pembrolizumab was successfully resumed as monotherapy after patient recovery, with no recurrence of PRES and continued disease control. There are other reports on monotherapy with variable efficacy. Matsuura et al. reported on a patient who resumed pembrolizumab monotherapy [15]. However, re-staging CT scans revealed multiple new metastases, and she was enrolled in a different clinical trial. Likewise, Abhishek et al. [13] reported their patient was successfully maintained on pazopanib monotherapy. This suggests that alternative systemic treatment strategies may remain feasible after resolution of lenvatinib-associated PRES, although decisions regarding rechallenge [18] or treatment modification [13] should be individualized. Although pembrolizumab has rarely been associated with PRES, its contribution in this case was considered unlikely given the close temporal relationship between lenvatinib initiation and symptom onset, as well as the absence of PRES recurrence after resumption of pembrolizumab monotherapy. This temporal association and clinical course are consistent with findings reported by Matsuura et al. [15] in which PRES occurring during lenvatinib and pembrolizumab combination therapy was attributed more strongly to lenvatinib exposure based on symptom onset following treatment initiation and tolerance of subsequent pembrolizumab monotherapy.

In our patient, the clinical and radiographic findings were consistent with the classical presentation of PRES, including acute onset of hypertension, encephalopathy, seizure, and bilateral parieto-occipital vasogenic edema on MRI [2,3]. What is particularly notable in this case is the rapid onset, with neurologic symptoms developing within two days of lenvatinib initiation, representing one of the earliest reported cases of lenvatinib-associated PRES in the literature. This rapid progression may reflect acute suppression of VEGF-mediated endothelial homeostasis, which, in the setting of concurrent severe hypertension, may have impaired cerebrovascular autoregulation and precipitated vasogenic edema [1,4]. The close temporal relationship between lenvatinib initiation and symptom onset, together with complete clinical and radiographic resolution following drug discontinuation, is consistent with lenvatinib as the likely precipitating agent. However, definitive causality cannot be established.

This case adds to the limited literature describing lenvatinib-associated PRES and, to our knowledge, represents a rare, documented instance in a patient with metastatic RCC. This population has been largely absent from prior reports, as summarized in Table 1, which has predominantly described PRES in the context of thyroid cancer and other malignancies. The rapid onset observed in our patient, within two days of lenvatinib initiation, is among the earliest reported in the cases summarized in Table 1 and suggests that clinical vigilance should begin immediately upon initiation of therapy rather than after weeks. Furthermore, the successful resumption of pembrolizumab monotherapy, with sustained disease control and no recurrence of PRES, offers a clinically meaningful example of how oncologic management can be adapted following this complication, providing practical guidance in an area where evidence remains scarce. As VEGFR tyrosine kinase inhibitors are increasingly used in the management of RCC, clinicians may encounter this toxicity more frequently, and cases such as this one are essential for building the clinical framework needed for early recognition, prompt management, and informed treatment decision-making.

## Conclusions

This case highlights the importance of recognizing PRES as a rare but serious potential complication of lenvatinib therapy. Practical recommendations for clinicians initiating lenvatinib include optimizing blood pressure control prior to treatment, closely monitoring during the first weeks of therapy, and educating patients to report headache, visual symptoms, confusion, or seizures urgently, with prompt brain MRI and drug discontinuation if PRES is suspected. Our patient's complete clinical and radiographic recovery, together with ongoing successful continuation of pembrolizumab monotherapy, illustrates that favorable neurologic and oncologic outcomes are achievable with early recognition and appropriate management. As VEGFR tyrosine kinase inhibitors become more widely used in metastatic RCC, this case contributes to the growing clinical framework for recognizing, managing, and adapting treatment following this rare but important complication.

## References

[1] Tseng YJ, Chen CN, Hong RL, Kung WM, Huang AP (2022). Posterior reversible encephalopathy syndrome after lenvatinib therapy in a patient with olfactory neuroblastoma. Brain Sci.

[2] Geocadin RG (2023). Posterior reversible encephalopathy syndrome. N Engl J Med.

[3] Covarrubias DJ, Luetmer PH, Campeau NG (2002). Posterior reversible encephalopathy syndrome: prognostic utility of quantitative diffusion-weighted MR images. AJNR Am J Neuroradiol.

[4] Mourid MR, Oweidat M, Abady E (2026). Posterior reversible encephalopathy syndrome (PRES): a narrative review of pathophysiology, clinical insights, and advances in management. Int J Emerg Med.

[5] Motzer R, Alekseev B, Rha SY (2021). Lenvatinib plus pembrolizumab or everolimus for advanced renal cell carcinoma. N Engl J Med.

[6] Simons M, Gordon E, Claesson-Welsh L (2016). Mechanisms and regulation of endothelial VEGF receptor signalling. Nat Rev Mol Cell Biol.

[7] Rathmell WK, Rumble RB, Van Veldhuizen PJ (2022). Management of metastatic clear cell renal cell carcinoma: ASCO guideline. J Clin Oncol.

[8] Faruque LI, Lin M, Battistella M (2014). Systematic review of the risk of adverse outcomes associated with vascular endothelial growth factor inhibitors for the treatment of cancer. PLoS One.

[9] Je Y, Schutz FA, Choueiri TK (2009). Risk of bleeding with vascular endothelial growth factor receptor tyrosine-kinase inhibitors sunitinib and sorafenib: a systematic review and meta-analysis of clinical trials. Lancet Oncol.

[10] (2009). AJCC Cancer Staging Handbook: From the AJCC Cancer Staging Manual. 7th ed. Springer: New York, NY.

[11] Chae YK, Chiec L, Adney SK (2018). Posterior reversible encephalopathy syndrome and takotsubo cardiomyopathy associated with lenvatinib therapy for thyroid cancer: a case report and review. Oncotarget.

[12] Osawa Y, Gozawa R, Koyama K, Nakayama T, Sagoh T, Sunaga H (2018). Posterior reversible encephalopathy syndrome after lenvatinib therapy in a patient with anaplastic thyroid carcinoma. Intern Med.

[13] Abhishek M, Renuka A, Ujjwal A (2022). Atypical posterior reversible encephalopathy syndrome associated with Lenvatinib therapy in a patient with metastatic thyroid cancer-a case report. Cancer Rep (Hoboken).

[14] Buenaventura DC, Vargas-Sierra H, Aristizabal-Henao N, Torres-Grajales JL, Aguilar-Londono C, Gutierrez-Restrepo J (2023). Posterior reversible encephalopathy syndrome as an adverse effect of lenvatinib in a patient with papillary thyroid carcinoma: a case report. Int J Endocrinol Metab.

[15] Matsuura Y, Nishida H, Kosaka T (2022). Case report: Posterior reversible encephalopathy syndrome, an adverse effect of lenvatinib and pembrolizumab combination therapy, in a patient with advanced endometrial cancer. Front Oncol.

[16] Chen M, Shen J, Jia R (2025). Case Report: Posterior reversible encephalopathy syndrome after lenvatinib treatment for hepatocellular carcinoma. Front Pharmacol.

[17] Velasco R, Villagrán M, Jové M (2021). Encephalitis induced by immune checkpoint inhibitors: a systematic review. JAMA Neurol.

[18] Fugate JE, Hawkes MA, Rabinstein AA (2025). Posterior reversible encephalopathy syndrome: evolving insights in diagnosis, management, and outcomes. Lancet Neurol.

[19] Moreau KL, Hildreth KL, Meditz AL, Deane KD, Kohrt WM (2012). Endothelial function is impaired across the stages of the menopause transition in healthy women. J Clin Endocrinol Metab.

